# Interpretable depression assessment using a large language model

**DOI:** 10.1371/journal.pdig.0001205

**Published:** 2026-02-09

**Authors:** Jae-Joong Lee, Jihoon Han, Choong-Wan Woo

**Affiliations:** 1 Center for Neuroscience Imaging Research, Institute for Basic Science, Suwon, South Korea; 2 Department of Biomedical Engineering, Sungkyunkwan University, Suwon, South Korea; 3 Department of Intelligent Precision Healthcare Convergence, Sungkyunkwan University, Suwon, South Korea; 4 Department of Brain Science and Engineering, Sungkyunkwan University, Suwon, South Korea; Xiangtan Central Hospital, CHINA

## Abstract

Detecting depression from conversational text using large language models (LLMs) has garnered significant interest. However, the limited interpretability of existing methods presents a major challenge for clinical application. To address this, we propose a novel framework for automatic depression assessment, which employs LLM prompting to extract interpretable factors linked to depression from text and uses linear regression to predict severity scores. We evaluated our approach using a benchmark dataset (DAIC-WOZ; *n* = 186), predicting Patient Health Questionnaire (PHQ)-8 scores from clinical interview transcripts. Our method identifies key behavioral and linguistic features indicative of depression while also achieving state-of-the-art performance with a mean absolute error (MAE) of 2.91 on the test set. The resulting model further generalizes to an independent test dataset (E-DAIC; *n* = 86) with an MAE of 2.86. These findings suggest that interpretable LLM-based approaches hold significant promise for enhancing the clinical utility of automated depression assessment.

## Author summary

Depression is a common and serious mental health concern, and there is a growing need to develop fast and accessible screening tools. Recently, detecting depression from conversational texts using large language models (LLMs) has emerged as a promising solution. However, most LLM-based methods operate as “black-box” models that provide little insight into how decisions are made, limiting their use in clinical settings. In this study, we propose a novel framework to enhance the interpretability of LLM-based depression assessment. Rather than asking an LLM to provide a single overall assessment, we prompt it to evaluate a set of specific depression-related factors in the text, spanning clinical symptoms, linguistic patterns, and cognitive distortions. These factor scores are then used in a linear regression model to predict depression severity, enabling a transparent understanding of which features contribute to the prediction. When evaluated on a benchmark clinical interview dataset, our method achieves state-of-the-art performance while also identifying key behavioral and linguistic markers of depression. Moreover, the resulting model further generalizes to an independent test dataset. These findings suggest that interpretable LLM-based approaches hold significant promise for enhancing the clinical utility of automated depression assessment.

## Introduction

Depression is a major mental health issue. In the United States, approximately one in five adults has been diagnosed with depression during their lifetime [1], and the annual economic cost is estimated to reach hundreds of billions of dollars [2]. Early detection and intervention are critical for improving clinical outcomes and reducing this burden [3]. However, traditional diagnostic methods, which rely heavily on clinician-administered interviews, are often time-intensive, resource-demanding, and inaccessible to many individuals with depression [4]. These challenges have prompted the need for automated assessment tools to support mental health screening in a fast, cost-efficient, and accessible manner [5,6].

Recent advances in large language models (LLMs) have opened new opportunities for addressing this need. LLMs, which are artificial intelligence systems trained on vast amounts of text data, demonstrate remarkable capabilities in understanding and processing natural language [7,8]. These advantages make LLMs well-suited for automated assessment of mental health conditions including depression, which are primarily diagnosed and treated through language [9–13]. A growing body of research has leveraged LLMs to detect depression from text data, such as social media posts [14–18] and clinical interview transcripts [19–30], with promising performances.

Despite these potentials, the limited interpretability of LLMs presents a significant challenge for clinical application. LLMs are highly complex models that operate with billions of parameters, making it difficult to understand the reasoning behind their outputs [11,31,32]. This “black box” nature is particularly concerning in healthcare settings, where clinicians need to understand how these models arrive at their decisions to identify potential errors and ensure responsible, trustworthy practice [12,32–34]. While previous studies have attempted to address this issue by querying LLMs to generate explanations for their own decisions [14–17], these self-explanations often lack faithfulness [35], can be misleading [36], and may only partially reflect the underlying reasoning process [37].

**Fig 1:**
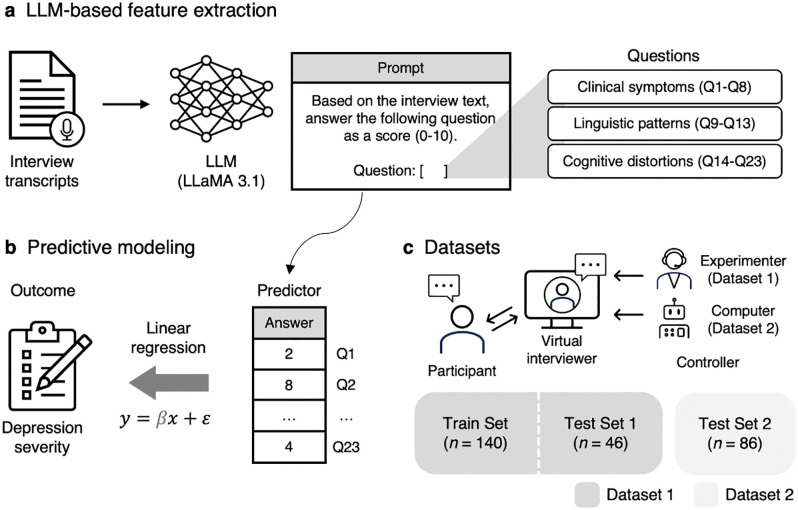
Overview of the proposed framework. **(a)** We use an LLM to extract interpretable features from interview transcripts. The task is to answer a set of questions, which have been associated with depression in previous literature, on a scale ranging from 0 to 10. The exact prompt used is provided in the Methods section. **(b)** The LLM-generated responses serve as predictors in a multiple linear regression model, estimating self-reported depression severity scores. **(c)** The framework was evaluated on two benchmark datasets that include semi-structured clinical interviews with a virtual interviewer, controlled by either a human experimenter (Dataset 1, DAIC-WOZ) or a fully autonomous computer agent (Dataset 2, E-DAIC). A subset of Dataset 1 (Train Set, *n* = 140) was used for model training, while the remaining subset of Dataset 1 (Test Set 1, *n* = 46) and all of Dataset 2 (Test Set 2, *n* = 86) were used for evaluation.

In this study, we propose a novel framework to enhance the interpretability of LLM-based depression assessment (Fig 1). Our approach does not rely solely on LLM for the entire assessment process. Rather, we use an LLM to extract a set of factors associated with depression in previous literature, spanning dimensions of clinical symptoms [38], linguistic patterns [39], and cognitive distortions [40]. These domain knowledge-informed factors then serve as features in a linear regression model to predict depression severity scores, allowing for direct interpretation of the factors contributing to the depression.

We evaluated our approach using the benchmark dataset for depression assessment, Distress Analysis Interview Corpus Wizard-of-Oz (DAIC-WOZ, Dataset 1) [41], which is a collection of 186 semi-structured clinical interviews with a human-controlled virtual interviewer. Our approach achieved state-of-the-art performance in predicting Patient Health Questionnaire (PHQ)-8 [38] depression severity scores from interview transcripts, while also identifying key features linked to depression from the regression model. The resulting model further demonstrated generalizability for an extended version of DAIC-WOZ (E-DAIC, Dataset 2 [41,42]), which consists of 86 interviews conducted by a fully autonomous computer agent. Overall, these findings highlight that our framework offers enhanced predictive accuracy and deepens our understanding of depression through interpretable predictions, thereby advancing the clinical utility of automated depression assessment.

## Results

### Interpretable LLM-derived features for depression assessment

Our LLM prompting strategy successfully generated a set of interpretable features from interview transcripts (S1 Fig). These features spanned three key domains informed by prior literature—clinical symptoms [38] (Q1-Q8), linguistic patterns [39] (Q9-Q13), and cognitive distortions [40] (Q14-Q23), allowing for a comprehensive and multidimensional assessment of depression (see Table 1 for the full list of questions used in LLM prompting).

**Table 1:** Questions used in LLM prompting.

ID	Question items
*Clinical symptoms*	
Q1	How much does Participant express having little interest or pleasure in doing things?
Q2	How much does Participant express feeling down, depressed, irritable or hopeless?
Q3	How much does Participant express trouble falling or staying asleep, or sleeping too much?
Q4	How much does Participant express feeling tired or having little energy?
Q5	How much does Participant express poor appetite or overeating?
Q6	How much does Participant express feeling bad about themselves – or that they are a failure or have let themselves or their family down?
Q7	How much does Participant express trouble concentrating on things, such as school work, reading or watching television?
Q8	How much does Participant express moving or speaking so slowly that other people could have noticed? Or the opposite – being so fidgety or restless that they have been moving around a lot more than usual?
*Linguistic patterns*	
Q9	How positive is Participant’s sentiment?
Q10	How negative is Participant’s sentiment?
Q11	To what extent is Participant’s language self-focused compared to other-focused (e.g., “I” vs. “They”)?
Q12	To what extent is Participant’s language present-focused compared to past- or future-focused (e.g., “I’m” vs. “I used to”)?
Q13	How effectively does Participant differentiate between similar emotions with distinct nuances (e.g., “sad” vs. “disappointed”)?
*Cognitive distortions*	
Q14	(Mindreading) To what extent does Participant assume others are thinking negatively about them without sufficient evidence? (e.g., “My boss hasn’t replied to the email I sent about the project days ago. He must think I’m incompetent.”)
Q15	(Catastrophizing) To what extent does Participant make negative predictions about the future without sufficient evidence? (e.g., “My boyfriend wants to spend more time with his friends. We’ll be distant and eventually break up.”)
Q16	(All-or-Nothing Thinking) To what extent does Participant view situations in extremes, without considering middle ground? (e.g., “I got a B+ on the exam, not an A. I’m a failure.”)
Q17	(Emotional Reasoning) To what extent does Participant believe something is true because it feels that way, even when the evidence suggests otherwise? (e.g., “My friends couldn’t get enough tickets for the concert. I know they didn’t mean to exclude me, but I feel rejected and believe they did.”)
Q18	(Labeling) To what extent does Participant assign negative labels to themselves based on specific incidents? (e.g., “I asked a woman to dance and she turned me down. I am a loser.”)
Q19	(Mental Filter) To what extent does Participant focus only on negative details, ignoring positive aspects? (e.g., “My boyfriend said I’m smart and fun, but also mentioned I’m demanding. I’m fixating on that comment and feeling bad.”)
Q20	(Overgeneralization) To what extent does Participant assume that one negative event will lead to a pattern of failures? (e.g., “I failed my math exam. I’ll probably fail the exams in my other courses as well.”)
Q21	(Personalization) To what extent does Participant assume personal responsibility for negative events that aren’t their fault? (e.g., “My company didn’t get the important contract. It must be my fault.”)
Q22	(Should Statements) To what extent does Participant think that things should or must be a certain way? (e.g., “I should always get at least a 90 on my exams. I’m upset because I got an 85.”)
Q23	(Minimizing or Disqualifying the Positive) To what extent does Participant ignore the positive things that happen to them? (e.g., “My boss said I did a great job on the sale, but I just got lucky with that. It wasn’t really because of my skill.”)

When we compared these features with their established reference metrics, where available, seven out of eight clinical symptom features showed significant correlation with the corresponding PHQ-8 items (S2 Fig), and three out of four linguistic pattern features with the relevant Linguistic Inquiry and Word Count (LIWC) [43]-based metrics (S3 Fig). This suggests that these features accurately reflected the underlying constructs they purported to measure. Furthermore, the majority of the LLM-derived features (20 out of 23) showed significant associations with depression severity in simple regression analyses (S4 Fig), suggesting that these features captured depression-relevant constructs.

### Prediction performance of the proposed framework

**Fig 2:**
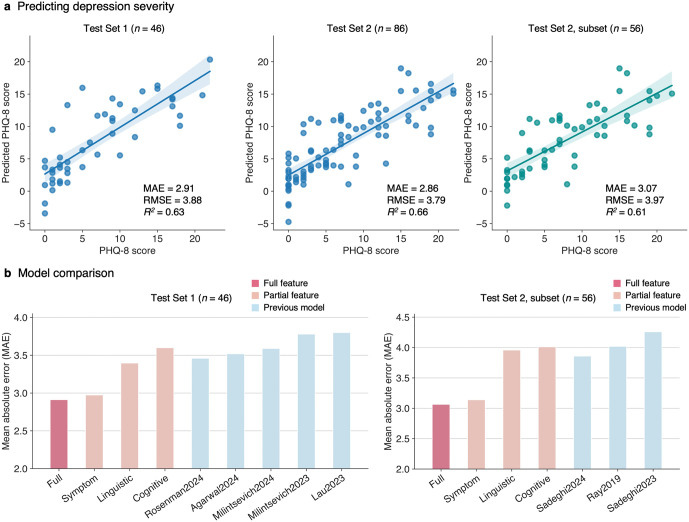
Prediction performance of the proposed framework. **(a)** Actual versus predicted PHQ-8 scores for Test Set 1 (left), Test Set 2 (middle), and a subset of the Test Set 2 used in prior studies (right), using the model trained with all LLM-derived features. Dots represent each participant’s data, with lines and shaded areas representing linear regression fit and the 95% confidence intervals, respectively. Performance metrics, including mean absolute error (MAE), root mean squared error (RMSE), and coefficient of determination (*R*^*2*^), are reported in each panel. **(b)** Comparisons of MAEs across models using all features (red), individual feature categories (pink), and previous models for text-based depression assessment (blue), evaluated on Test Set 1 (left) and the subset of Test Set 2 (right).

Our framework demonstrated state-of-the-art performance in predicting depression severity from interview text (Fig 2). The regression model using all LLM-derived features achieved a mean absolute error (MAE) of 2.91 on Test Set 1 (*n* = 46), outperforming previous text-based depression assessment models (MAEs = 3.46 [29], 3.52 [44], 3.59 [45], 3.78 [46], 3.80 [47]). This model further showed strong generalizability for Test Set 2 (*n* = 86) with an MAE of 2.86, which is comparable to the results on Test Set 1 despite the differences in the interview condition (i.e., human- vs. computer-controlled). When compared against prior models evaluated on a subset of Test Set 2 (*n* = 56 out of 86), our model achieved an MAE of 3.07, outperforming the previous benchmarks (MAEs = 3.86 [24], 4.02 [48], 4.06 [23]).

Models trained on individual feature categories also demonstrated robust prediction performance (Fig 2b). The model using clinical symptom-related features alone achieved an MAE of 2.97 on Test Set 1 and 3.14 on the subset of Test Set 2, outperforming all previous models. Interestingly, the models using linguistic patterns or cognitive distortion features, which are not part of the PHQ framework, still demonstrated competitive results with MAEs of 3.40 and 3.60 on Test Set 1 and 3.96 and 4.01 on the subset of Test Set 2, highlighting the value of including features indirectly related to depression.

Comparison with the two alternative prompting strategies further validated the specificity and effectiveness of our approach (S5 Fig). When the model was prompted with non-depression-related questions (NQ1-NQ10; S1 Table), prediction performance significantly degraded, with an MAE of 5.12 on Test Set 1 and 5.45 on Test Set 2, emphasizing the importance of using domain-guided features over generic comprehension features. Moreover, directly asking an LLM to provide depression severity scores (DQ1; S1 Table) did not yield improved prediction performance over our framework, with an MAE of 3.51 on Test Set 1 and 3.87 on Test Set 2, indicating that our framework outperforms end-to-end depression assessment in both accuracy and transparency.

To further examine the robustness of our prompting procedure, we tested an alternative LLM parameter configuration by switching from the deterministic greedy-search setting (“top_p” = 0, “temperature” = 0.0001) to a probabilistic setting (“top_p” = 0.8, “temperature” = 0.7) to allow more variable and stochastic responses across runs. We conducted 10 iterations of LLM response generation under this stochastic configuration (S6 Fig). Although the probabilistic configuration led to slightly degraded performance, our framework still outperformed previous text-based depression assessment models across all iterations (MAEs = 2.85-3.45 for Test Set 1, 2.98-3.59 for Test Set 2, and 3.24-3.85 for the subset of Test Set 2). Moreover, we examined the impact of prompt wording variation by instructing the LLM to generate an alternative version of the original prompt and re-running the response generation with this modified prompt (S7 Fig). The model showed prediction accuracies comparable to the original results and continued to outperform prior models (MAEs = 3.22, 3.31, and 3.36 for Test Set 1, Test Set 2, and the subset of Test Set 2, respectively). These findings demonstrate the robustness of the proposed framework to stochastic LLM parameter configurations and variations in prompt wording.

We additionally tested more complex, nonlinear algorithms, including support vector regression (SVR) with a radial basis function (RBF) kernel (S8 Fig) and an ensemble learning approach that combined predictions from models trained on individual feature categories (S9 Fig). Neither method outperformed linear regression on either Test Set 1 (MAEs = 3.44 for SVR and 3.02 for ensemble learning) or Test Set 2 (MAEs = 3.30 for SVR and 3.23 for ensemble learning), suggesting that the simple linear model was sufficient to capture the key relationships between features and depression severity without the need for more complex models.

Lastly, we examined whether employing a medical domain fine-tuned LLM could further improve performance (S10 Fig). The medical LLM-based model achieved higher predictive accuracy on Test Set 1 (MAE = 2.59) but showed reduced generalizability on Test Set 2 (MAE = 3.29) and its subset (MAE = 3.45). These findings suggest that while medical fine-tuning may enhance in-domain performance, a general-purpose LLM provides greater robustness and generalizability across diverse interview contexts.

### Feature contributions to depression severity prediction

**Fig 3:**
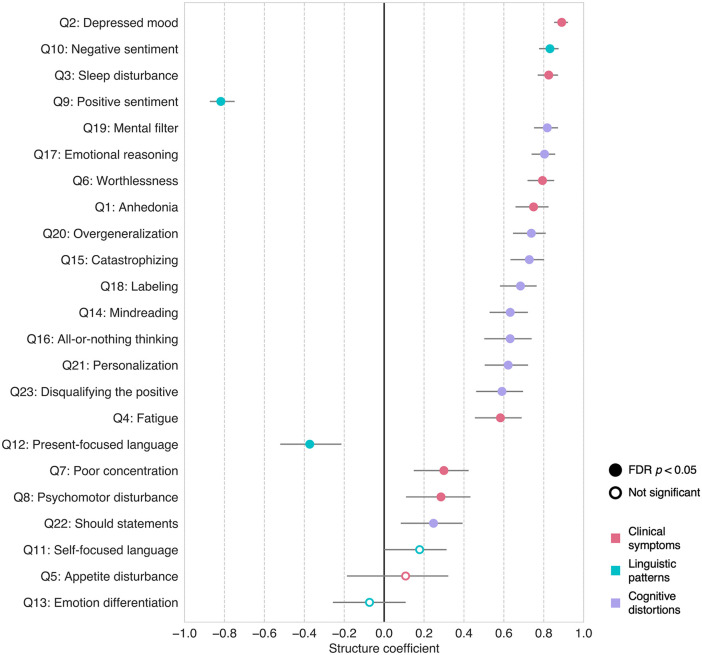
Structure coefficients of the multiple regression model predicting depression severity. The plot presents structure coefficients (x-axis), which represent the correlation between individual LLM-derived features (y-axis) and the model’s predicted outcome, across all Test Set data (*n* = 132). Error bars represent 95% confidence intervals calculated via bootstrap sampling with 10,000 iterations. Marker shapes indicate statistical significance: false discovery rate (FDR)-corrected *p* < 0.05 (filled circles) and not significant (open circles). Marker colors represent feature categories: red, clinical symptoms; cyan, linguistic patterns; and purple, cognitive distortions. Features are ordered top to bottom by the absolute magnitude of their structure coefficients.

Our framework allows for the evaluation of the relative contributions of LLM-derived features (Q1-Q23) to the prediction of depression severity in any given test dataset. In this study, we used structure coefficients [49], an established indicator of feature contributions in statistics literature [50,51]. This metric is known to be particularly useful in the presence of multicollinearity, which can obscure the interpretation of multiple regression coefficients and ablation-based feature importance (see the correlation matrix among features in S11 Fig; regression coefficients in S12 Fig; and ablation feature importance in S13 Fig). Fig 3 displays structure coefficients calculated from the combined Test Sets (*n* = 132). We also present structure coefficients separately for Test Set 1 and 2 in S14 and S15 Figs, but the results are largely consistent with those from the combined data.

Most of the LLM-derived features (i.e., 20 out of 23) showed significant structure coefficients, suggesting their collective contribution to the prediction of depression severity. To aid interpretation, we examined the top five features with the largest structure coefficients that span all three feature categories. First, clinical symptoms including depressed mood (Q2: *r* = 0.89, 95% confidence interval [CI] = 0.85 to 0.92) and sleep disturbance (Q3: *r* = 0.82, 95% CI = 0.77 to 0.87) exhibited the largest and third-largest structure coefficients, respectively. These features reflect the core affective and somatic symptoms of depression [52] and are among the most robust discriminators between depressed and non-depressed individuals [53] in the literature. Linguistic patterns also emerged as important predictors, with negative sentiment (Q10: *r* = 0.83, 95% CI = 0.78 to 0.87) and positive sentiment (Q9: *r* = -0.82, 95% CI = -0.87 to -0.75) exhibiting the second- and fourth-largest structure coefficients, respectively. These sentiment features reflect the overall emotional tone of language [39] and are increasingly recognized as linguistic markers of depression [54,55]. Lastly, in the cognitive distortion category, mental filter (Q19: *r* = 0.82, 95% CI = 0.75 to 0.87) ranked fifth. This distortion, characterized by a biased focus on negative information [40], is commonly observed in individuals with depression [56,57], and its modification is associated with reductions in depression severity [58].

Overall, these results highlight the distinct contributions of depression-relevant features to depression severity prediction, underscoring the potential of our framework to identify key behavioral and linguistic markers, such as depressed mood, sleep disturbance, language sentiment, and mental filtering.

## Discussion

In this study, we introduced an interpretable, LLM-based framework for automated depression assessment. Leveraging domain knowledge-informed feature extraction combined with linear regression, our framework achieved state-of-the-art performance on two benchmark datasets for automated depression assessment, DAIC-WOZ (Test Set 1, MAE = 2.91) and E-DAIC (Test Set 2, MAE = 2.86), surpassing previous text-based models. Analysis of the structure coefficients provided direct insights into the relative importance of each feature, highlighting depressed mood, sleep disturbance, language sentiment, and mental filtering as key indicators of depression. These findings suggest that our framework improves both the accuracy and interpretability of automated depression assessment, supporting its potential clinical utility.

A central contribution of the proposed framework lies in addressing the interpretability limitations of prior LLM-based approaches to mental health assessment. LLMs often function as opaque “black-box” models; thus, relying solely on LLMs for complex clinical judgments, such as predicting depression severity, limits both interpretability and practical utility [11,12,31–34]. Our framework mitigates this issue by leveraging LLMs’ natural language processing capabilities for simpler, well-defined feature extraction tasks, rather than full end-to-end severity prediction. While previous studies have also explored LLM-based feature extraction related to depression [27–29], these efforts primarily focused on improving predictive accuracy and were limited to a narrow set of features. In contrast, our framework employs a systematically curated set of features grounded in domain knowledge to capture a broad spectrum of depression-related dimensions, enabling interpretable, multidimensional representations of depression. Although this study focused on the pre-defined set of 23 features, researchers can examine alternative feature sets by modifying the prompting questions, allowing them to explore different dimensions of depression aligned with their specific hypotheses.

We demonstrated that most of the LLM-derived features aligned with their established reference metrics, where available, supporting the overall validity of our feature extraction process. However, there were also exceptions, including psychomotor disturbance (Q8) and self-focused language (Q11), as indicated by null relationships shown in S2 and S3 Figs. Since psychomotor disturbance is difficult to assess from text alone, especially when not explicitly mentioned by participants, future work may integrate multimodal features such as audio or video data to improve the detection of such behavioral symptoms [24,48]. For self-focused language, the LLM-generated scores exhibited minimal variability (e.g., 137 of 140 samples were scored as “8”), indicating a need to increase sensitivity to this feature by refining prompt design or model architecture—for instance, by incorporating examples of interview text with corresponding scores within the prompt (i.e., in-context learning) or through fine-tuning approaches.

Interestingly, our framework is designed with a focus on interpretability but also outperformed prior approaches that prioritized predictive accuracy. This suggests that breaking down the complex task of depression severity prediction into smaller, interpretable steps may enhance overall model performance. Specifically, our multi-stage process—consisting of feature extraction followed by subsequent linear regression—may benefit from a form of structured reasoning akin to the “chain-of-thought” prompting, which has been shown to improve complex problem-solving in LLMs [59]. Moreover, by embedding domain knowledge into the feature extraction process, our approach may help prioritize clinically relevant signals while reducing noise from unrelated linguistic patterns [16,18]. Together, these results highlight that interpretability and accuracy need not be competing goals, and that careful system design can advance both dimensions simultaneously.

Our use of linear regression, combined with structure coefficients, enables each prediction to be decomposed into contributions from interpretable, domain knowledge-informed constructs. This transparency allows clinicians to understand how the model arrives at its decisions—an essential requirement for responsible deployment in healthcare settings [12,32–34]. As expected, core depressive symptoms such as depressed mood and sleep disturbance emerged among the most influential predictors, aligning with established clinical understanding [52,53]. Notably, several linguistic patterns and cognitive distortion features, such as language sentiment and mental filter, also showed strong contributions to predicted severity. While recent studies have explored their associations with depression [54–58], their predictive utility remains relatively underexplored, to our knowledge. Our findings suggest that such features may provide additional information beyond what is captured by traditional clinical symptomatology. The ability of our framework to detect these subtle linguistic patterns or cognitive distortions from text highlights its promise for early screening and intervention, particularly in contexts where conventional clinical assessments are less accessible. We also found some features with non-significant structure coefficients, such as Q5, Q11, and Q13, which showed weak, non-significant correlations with most of the other features. However, since the structure coefficients were derived using held-out test data, we retained the full feature set, as removing features on this basis would constitute an inherently post-hoc decision and could introduce bias. Future studies may explore principled feature selection approaches using additional independent training data to further refine the feature set while avoiding post-hoc bias.

This study has several limitations. First, while our framework demonstrated strong performance on semi-structured clinical interviews, its generalizability to other types of text data—such as social media posts, everyday conversations, or telehealth interactions—has yet to be established. Second, we did not demonstrate the validity of LLM-derived features for emotion differentiation (Q13) and cognitive distortions (Q14-Q23), as no reference metrics were available for these constructs. In addition, we did not provide clinical validity evidence for the features, such as through a blinded evaluation by clinical experts. Thus, future studies could expand the collection of reference metrics, for example, through expert annotations or other clinically grounded assessments [44,60], to enable more rigorous validation and further enhance interpretability. Finally, our set of 23 features may not fully capture the multifaceted nature of depression. Expanding this feature set to include additional clinically relevant variables could enhance the model’s effectiveness.

In summary, we present an interpretable framework for depression assessment that integrates LLM-based feature extraction with linear regression. Our findings highlight the potential of interpretable LLM-based approaches to enhance both prediction accuracy and interpretability, offering a promising path toward clinically useful automated mental health assessment.

## Materials and methods

### Ethics statement

The datasets used in this study (i.e., DAIC-WOZ [41] and E-DAIC [41,42]) are publicly available (https://dcapswoz.ict.usc.edu/) and were originally collected with approval from the University of Southern California Institutional Review Board (UP-11–00342) [61]. All participants provided informed consent, and the interviews were de-identified prior to public release. As only fully anonymized data were used in this study, no additional ethical approval was required for our analysis.

### Datasets

This study used Dataset 1 to investigate the proposed framework, and Dataset 2 to test the generalizability of the framework (Fig 1c). Dataset 1 (DAIC-WOZ) [41] is a collection of semi-structured clinical interviews, and has been widely used as a benchmark for automatic mental health assessment. Participants were recruited from the general public and U.S. veterans living in the Greater Los Angeles metropolitan area. During the interviews, participants sat alone in a room and interacted with “Ellie,” a virtual interviewer displayed on a computer screen and controlled by a human experimenter in a separate room. Each interview was conducted in English for 5–20 minutes, beginning with neutral questions to build rapport, progressing to specific questions about symptoms and events related to depression and post-traumatic stress, and ending with cool-down questions designed to ensure participants did not leave in distress. The dataset consists of 189 participants’ interview recordings and transcripts, along with their depression severity scores assessed using the Patient Health Questionnaire (PHQ)-8 [38]. Three participants were excluded due to missing interviewers’ text in transcripts, resulting in a final sample of 186 participants. Following a pre-defined data split, we assigned 140 samples to the Train Set (age = 38.0 ± 12.1 [mean ± SD], 61 female) and 46 samples to Test Set 1 (age = 41.6 ± 13.1 [mean ± SD], 23 female). The distribution of PHQ-8 scores for both sets is shown in S16 Fig.

Dataset 2 (E-DAIC) [41,42] is an extended version of the DAIC-WOZ. Procedures to collect the interview were the same, except that the virtual interviewer “Ellie” was controlled by a fully autonomous computer agent. The dataset consists of 86 participants’ interview recordings and transcripts, along with their PHQ-8 depression severity scores. Unlike the DAIC-WOZ, the provided transcripts of the E-DAIC contained substantial errors, possibly due to the reliance on the Google Cloud’s speech-to-text processing without additional corrections. To improve accuracy, we re-transcribed the interviews in the following procedures. First, we used OpenAI’s Whisper [62] large-v3 model for automatic transcription from interview recordings. Then, these transcriptions were reviewed and manually corrected by two English-fluent researchers. Finally, these transcriptions were double-checked and refined by the other researcher to ensure quality. We used the whole 86 samples (age = 46.0 ± 11.9 [mean ± SD], 19 female) as Test Set 2. The distribution of PHQ-8 scores is shown in S16 Fig.

### LLM-based feature extraction

The first step of our framework is to extract a set of interpretable, depression-related factors from interview transcripts using zero-shot LLM prompting (Fig 1a). We employed Meta’s LLaMA 3.1-70B-Instruct model [63] (https://huggingface.co/meta-llama/Llama-3.1-70B-Instruct), a high-performing open-source, instruction-tuned model selected for its reproducibility, transparency, and privacy advantages over proprietary LLMs. The exact prompt used in this study is as follows.

*The following text is a semi-structured clinical interview conducted by the virtual interviewer “Ellie” with interviewee “Participant” with varying depressive symptoms.*

*[Interview]*

*[INSERT_INTERVIEW]*

*[End of interview]*

*Based on the interview text, answer the following question.*

*Question: [INSERT_QUESTION]*

*Answer should be a score between 0 and 10, where 0 means “Not at all” and 10 means “Extremely”. Return only the score.*

In this prompt, the interview transcript is inserted into *[INSERT_INTERVIEW]*, while the question being assessed is placed in *[INSERT_QUESTION]*. To ensure a single, deterministic response from the prompting, we used a deterministic greedy-search strategy by configuring the parameters “top_p” to 0, “temperature” to 0.0001, and “max_new_tokens” to 1.

We designed the questions used for prompting to assess factors associated with depression in previous literature (Table 1). The questions fall into three categories.

1. Clinical symptoms (Q1-Q8): These questions were adapted from the PHQ-8 [38] to assess the clinical symptoms of depression, each item of which aligns with one of the Diagnostic and Statistical Manual of Mental Disorders (DSM)-5 [64] criteria for major depressive disorder. The questions include (Q1) diminished interest or pleasure, (Q2) depressed mood, (Q3) insomnia or hypersomnia, (Q4) fatigue or loss of energy, (Q5) poor appetite or overeating, (Q6) feelings of worthlessness or guilt, (Q7) diminished ability to think or concentrate, and (Q8) psychomotor agitation or retardation.
2. Linguistic patterns (Q9-Q13): These questions assess the linguistic patterns which have been linked with depression [39], including sentiment, linguistic focus, and emotion differentiation. Sentiment refers to the overall emotional tone, including (Q9) positive sentiment and (Q10) negative sentiment, and can be related to subjective depressive mood [54,55]. Linguistic focus includes social and temporal focus, assessed by the relative use of (Q11) self-focused language compared to other-focused one (e.g., “I” vs. “They”) and (Q12) present-focused language compared to past- or future-focused one (e.g., “I’m” vs. “I used to”). Shifting of word use to be psychologically distant terms (i.e., other-focused language or past- or future-focused language) has been considered as an emotion regulation strategy [65] and is associated with reduced depressive symptoms [66], whereas prior literature also suggests that non-present, particularly past-focused language is a key marker of depression [67–70]. Emotion differentiation refers to (Q13) the ability to differentiate between similar emotions with distinct nuances (e.g., “sad” vs. “disappointed”), the impairment of which has been associated with depression [71,72].
3. Cognitive distortions (Q14-Q23): These questions were adapted from the Cognitive Distortion Scale (CDS) [40] to assess the ten representative types of cognitive distortions, including (Q14) mindreading, (Q15) catastrophizing, (Q16) all-or-nothing thinking, (Q17) emotional reasoning, (Q18) labeling, (Q19) mental filter, (Q20) overgeneralization, (Q21) personalization, (Q22) should statements, and (Q23) minimizing or disqualifying the positive. These distortions are common in individuals with depression [56,57] and are a central focus of cognitive behavioral therapy (CBT) [73], a widely used treatment for depression.

We repeated the LLM prompting for each separate question to obtain a numeric score ranging from 0 to 10 for each factor, resulting in a set of interpretable, depression-relevant features.

### Evaluating feature relevance to depression

To assess whether the LLM-derived features are relevant to depression, we conducted univariate regression analysis using each LLM-derived feature as a predictor variable and PHQ-8 score as an outcome variable within Train Set (*n* = 140). Prior to model fitting, predictor variable was *z*-scored such that the resulting regression coefficients (*β*) can be compared across different LLM-derived features. We calculated 95% confidence intervals (CIs) and *p*-values of regression coefficients via bootstrap sampling with 10,000 iterations, and corrected the *p*-values for multiple comparisons using the Benjamini-Hochberg method to control the false discovery rate (FDR) across all the features (Q1-Q23).

### Evaluating the validity of feature extraction

To assess whether the LLM-derived features accurately reflected the underlying constructs they purported to measure, we compared these features to established reference metrics where available. For clinical symptom features (Q1-Q8), we calculated Spearman’s correlations between the LLM-generated scores and their corresponding item-level responses from the PHQ-8 (e.g., Q1 with PHQ-8 item 1, Q2 with PHQ-8 item 2, etc.). For the linguistic pattern features (Q9-Q12), we calculated Spearman’s correlations between the LLM-generated scores and the relevant metrics from the Linguistic Inquiry and Word Count (LIWC)-22 [43], a popular linguistic analysis tool. These LIWC metrics included (Q9) positive sentiment and (Q10) negative sentiment, representing relative frequency of words with positive sentiment (“*tone_pos*”) and negative sentiment (“*tone_neg*”), and (Q11) self-focused language and (Q12) present-focused language, representing the proportion of first-person pronouns (“*i*”, “*we*”) among all pronouns (“*i*”, “*we*”, “*you*”, “*shehe*”, “*they*”) and present-tense words (“*focuspresent*”) among all tense words (“*focuspresent*”, “*focuspast*”, “*focusfuture*”). We did not conduct quantitative comparisons for emotion differentiation (Q13) and the cognitive distortions (Q14-Q23), as no reference metrics were available for these constructs. All correlations were calculated within Train Set (*n* = 140), and *p*-values were corrected for multiple comparisons using the Benjamini-Hochberg method to control the FDR across the tested features (Q1-Q12).

### Predictive modeling

The next step of our framework is to predict the depression severity scores based on the domain knowledge-informed factors generated from the LLM. (Fig 1b). We employed multiple linear regression with the LLM-derived features as predictor variables and the PHQ-8 score as an outcome variable. To evaluate the predictive utility of different feature sets, we trained separate models using either all features combined or features from individual categories (i.e., clinical symptoms, linguistic patterns, and cognitive distortions). Prior to model fitting, all predictor variables were *z*-scored. We developed the model from Train Set (*n* = 140) and tested the model onto Test Set 1 (*n* = 46) and Test Set 2 (*n* = 86). Model performance was primarily assessed using mean absolute error (MAE), with root mean squared error (RMSE) and coefficient of determination (*R*^*2*^) also reported. For comparison with previous studies that used a subset of the Test Set 2 (*n* = 56 out of 86), we additionally reported model performances on this subset. We calculated 95% CIs and *p*-values of regression coefficients via bootstrap sampling with 10,000 iterations and corrected the *p*-values for multiple comparisons using the Benjamini-Hochberg method to control the FDR across all the features (Q1-Q23).

### Comparison with alternative prompting strategies

To assess the specificity and effectiveness of our domain knowledge-informed feature extraction approach, we conducted two additional control analyses using alternative sets of questions for comparison (S1 Table).

1. Non-depression-related questions (NQ1-NQ10): We first tested whether the predictive power of our framework stems from meaningful assessment of depression-related features rather than general text comprehension. To this end, we replaced the original depression-related questions with a set of control questions that explicitly focused on non-clinical topics (e.g., political opinions, musical interests, and food preferences). We then applied the same modeling framework to predict PHQ-8 scores based on these non-depression-related features.
2. Direct severity assessment (DQ1): We further tested a more simple, end-to-end use of an LLM by employing a single question to directly provide an overall severity score from text (“*How severe are Participant’s depressive symptoms?*”). We then applied the same modeling framework to map these LLM-generated overall severity estimates onto PHQ-8 scores, ensuring consistency with other prompting strategies.

## Supporting information

S1 Fig LLM-derived features for depression assessment. Each row represents one of the 23 questions used in LLM prompting, except for the final row (“Sev”, which stands for depression severity). “Sev” indicates the self-reported PHQ-8 scores normalized to a 0–10 scale. Each column corresponds to a participant in Train Set (*n* = 140), ordered by increasing PHQ-8 scores (“Sev”). Color represents the magnitude of both LLM-generated scores and normalized PHQ-8 scores.
(TIF)

S2 Fig Correspondence of clinical symptom features with PHQ-8 items. Each panel shows the LLM-generated scores for clinical symptoms (Q1-Q8) and the corresponding PHQ-8 item scores across participants in the Train Set (*n* = 140). Dots represent each participant, with colors indicating item scores (ranging from 0 to 3). Lines and shaded areas represent linear regression fit and the 95% confidence intervals, respectively. Spearman’s rank correlation coefficients and associated *p*-values are reported in each panel. All the *p*-values are corrected for multiple testing using the Benjamini-Hochberg method to control the false discovery rate (FDR) across all tested features (Q1-Q12).
(TIF)

S3 Fig Correspondence of linguistic pattern features with LIWC metrics. Each panel shows the LLM-generated scores for linguistic patterns (Q9-Q12) and the corresponding Linguistic Inquiry and Word Count (LIWC) scores across participants in the Train Set (*n* = 140). Dots represent each participant, and lines and shaded areas represent linear regression fit and the 95% confidence intervals. Spearman’s rank correlation coefficients and associated *p*-values are reported in each panel. All the *p*-values are corrected for multiple testing using the Benjamini-Hochberg method to control the false discovery rate (FDR) across all tested features (Q1-Q12).
(TIF)

S4 Fig Standardized beta coefficients of the simple regression model predicting depression severity. The plot presents standardized regression beta coefficients (x-axis) for individual LLM-derived features (y-axis). Error bars represent 95% confidence intervals calculated via bootstrap sampling with 10,000 iterations. Marker shapes indicate statistical significance: false discovery rate (FDR)-corrected *p* < 0.05 (filled circles) and not significant (open circles). Marker colors represent feature categories: red, clinical symptoms; cyan, linguistic patterns; and purple, cognitive distortions. Features are ordered top to bottom by the absolute magnitude of their regression coefficients.
(TIF)

S5 Fig Prediction performance of alternative prompting strategies. Comparisons of MAEs across models using three prompting methods: the proposed method using 23 depression-related questions (red), prompting with 10 non-depression-related questions (gray), and prompting with a direct severity assessment question (black). Results are shown for Test Set 1 (left) and Test Set 2 (right).
(TIF)

S6 Fig Prediction performance using stochastic configuration. Box plots show the distribution of mean absolute errors (MAEs) for Test Set 1 (left), Test Set 2 (middle), and a subset of the Test Set 2 used in prior studies (right), across 10 iterations of LLM response generation under the stochastic configuration (“top_p” = 0.8, “temperature” = 0.7). Each box spans from the first to the third quartile, with the horizontal line inside representing the median. Whiskers extend to the smallest and largest values within 1.5 times the interquartile range from the lower and upper quartiles. Dots represent data of each iteration.
(TIF)

S7 Fig Prediction performance under prompt wording variation. Actual versus predicted PHQ-8 scores for Test Set 1 (left), Test Set 2 (middle), and a subset of the Test Set 2 used in prior studies (right) under prompt wording variation. The LLM was instructed to generate a modified version of the original instruction (“Reword the following LLM prompt. The last sentence should be “Return only the score.” Return only the modified LLM prompt.”), and we subsequently re-evaluated the framework using this modified prompt. Dots represent each participant’s data, with lines and shaded areas representing linear regression fit and the 95% confidence intervals, respectively. Performance metrics, including mean absolute error (MAE), root mean squared error (RMSE), and coefficient of determination (*R*^*2*^), are reported in each panel.
(TIF)

S8 Fig Prediction performance of nonlinear support vector regression. Actual versus predicted PHQ-8 scores for Test Set 1 (left), Test Set 2 (middle), and a subset of the Test Set 2 used in prior studies (right), using support vector regression (SVR) with a radial basis function (RBF) kernel. The SVR hyperparameter *C* was optimized using leave-one-out cross-validation (LOOCV) on the Train Set (*n* = 140). Dots represent each participant’s data, with lines and shaded areas representing linear regression fit and the 95% confidence intervals, respectively. Performance metrics, including mean absolute error (MAE), root mean squared error (RMSE), and coefficient of determination (*R*^*2*^), are reported in each panel.
(TIF)

S9 Fig Prediction performance of stacking ensemble learning. Actual versus predicted PHQ-8 scores for Test Set 1 (left), Test Set 2 (middle), and a subset of the Test Set 2 used in prior studies (right), using a stacking ensemble model that combines predictions from models trained on individual feature categories. Predictions from each feature-category model were generated using leave-one-out cross-validation (LOOCV) on the Train Set (*n* = 140) to prevent overfitting. Dots represent each participant’s data, with lines and shaded areas representing linear regression fit and the 95% confidence intervals, respectively. Performance metrics, including mean absolute error (MAE), root mean squared error (RMSE), and coefficient of determination (*R*^*2*^), are reported in each panel.
(TIF)

S10 Fig Prediction performance using a medical domain fine-tuned LLM. Actual versus predicted PHQ-8 scores for Test Set 1 (left), Test Set 2 (middle), and a subset of the Test Set 2 used in prior studies (right), using Med42-v2-70B, a model built upon LLaMA 3-70B and fine-tuned on clinical datasets. Dots represent each participant’s data, with lines and shaded areas representing linear regression fit and the 95% confidence intervals, respectively. Performance metrics, including mean absolute error (MAE), root mean squared error (RMSE), and coefficient of determination (*R*^*2*^), are reported in each panel.
(TIF)

S11 Fig Feature correlation matrix. Colors represent Spearman correlation coefficients (*r*) between all LLM-derived features (Q1-Q23) in the Train Set (*n* = 140). Thresholding was applied at false discovery rate (FDR)-corrected *p* < 0.05.
(TIF)

S12 Fig Standardized beta coefficients of the multiple regression model predicting depression severity. The plot presents standardized regression beta coefficients (x-axis) for individual LLM-derived features (y-axis). Error bars represent 95% confidence intervals calculated via bootstrap sampling with 10,000 iterations. Marker shapes indicate statistical significance: false discovery rate (FDR)-corrected *p* < 0.05 (filled circles), uncorrected *p* < 0.05 (rhombus), and not significant (open circles). Marker colors represent feature categories: red, clinical symptoms; cyan, linguistic patterns; and purple, cognitive distortions. Features are ordered top to bottom by the absolute magnitude of their regression coefficients.
(TIF)

S13 Fig Ablation-based feature importance. The plot presents ablation-based feature importance values (*x*-axis), which represent the change in prediction accuracy (mean absolute error, MAE) after the removal of individual LLM-derived features (*y*-axis) across all Test Set data (*n* = 132). Error bars represent 95% confidence intervals calculated via bootstrap sampling with 10,000 iterations. Marker shapes indicate statistical significance: false discovery rate (FDR)-corrected *p* < 0.05 (filled circles) and not significant (open circles). Marker colors represent feature categories: red for clinical symptoms, cyan for linguistic patterns, and purple for cognitive distortions. Features are ordered from top to bottom by the absolute magnitude of their ablation feature importance values.
(TIF)

S14 Fig Structure coefficients of the multiple regression model predicting depression severity for Test Set 1. The plot presents structure coefficients (x-axis), which represent the correlation between individual LLM-derived features (y-axis) and the model’s predicted outcome, for Test Set 1 (*n* = 46). Error bars represent 95% confidence intervals calculated via bootstrap sampling with 10,000 iterations. Marker shapes indicate statistical significance: false discovery rate (FDR)-corrected *p* < 0.05 (filled circles) and not significant (open circles). Marker colors represent feature categories: red, clinical symptoms; cyan, linguistic patterns; and purple, cognitive distortions. Features are ordered top to bottom by the absolute magnitude of their structure coefficients.
(TIF)

S15 Fig Structure coefficients of the multiple regression model predicting depression severity for Test Set 2. The plot presents structure coefficients (x-axis), which represent the correlation between individual LLM-derived features (y-axis) and the model’s predicted outcome, for Test Set 2 (*n* = 86). Error bars represent 95% confidence intervals calculated via bootstrap sampling with 10,000 iterations. Marker shapes indicate statistical significance: false discovery rate (FDR)-corrected *p* < 0.05 (filled circles) and not significant (open circles). Marker colors represent feature categories: red, clinical symptoms; cyan, linguistic patterns; and purple, cognitive distortions. Features are ordered top to bottom by the absolute magnitude of their structure coefficients.
(TIF)

S16 Fig Distribution of PHQ-8 scores. Box plots show the distribution of PHQ-8 scores for Train Set (red, *n* = 140), Test Set 1 (brown, *n* = 46), and Test Set 2 (green, *n* = 86). Each box spans from the first to the third quartile, with the horizontal line inside representing the median. Whiskers extend to the smallest and largest values within 1.5 times the interquartile range from the lower and upper quartiles. Dots represent each participant’s data.
(TIF)

S1 Table Questions used for alternative LLM prompting strategies. (DOCX)
