# Supplementary material for: Interpretable depression assessment using a large language model
Source: PLOS Digit Health. 2026 Feb 9;5(2):e0001205. doi: 10.1371/journal.pdig.0001205 (PMC12885269; doi:10.1371/journal.pdig.0001205)
Supplement: S1 Table — (DOCX) [file pdig.0001205.s017.docx]

**S1 Table. Questions used for alternative LLM prompting strategies.**

| ID | Question items |
| --- | --- |
| *Non-depression-related questions* | |
| NQ1 | To what extent does Participant express thoughts on political issues? |
| NQ2 | To what extent is Participant interested in music or musical instruments? |
| NQ3 | To what extent does Participant have preferences for certain types of food or cuisine? |
| NQ4 | To what extent is Participant engaged in financial planning or budgeting? |
| NQ5 | To what extent does Participant use numerical information in their description? |
| NQ6 | To what extent is Participant working in technical or scientific fields compared to humanities or social sciences? |
| NQ7 | To what extent does Participant express religious beliefs? |
| NQ8 | To what extent does Participant enjoy watching movies or television shows? |
| NQ9 | To what extent does Participant have an interest in animals? |
| NQ10 | To what extent does Participant prefer urban living to rural living? |
| *Direct severity assessment question* | |
| DQ1 | How severe are Participant's depressive symptoms? |
